# Tumor Necrosis Factor-alpha utilizes MAPK/NFκB pathways to induce cholesterol-25 hydroxylase for amplifying pro-inflammatory response via 25-hydroxycholesterol-integrin-FAK pathway

**DOI:** 10.1371/journal.pone.0257576

**Published:** 2021-09-22

**Authors:** Swechha M. Pokharel, Kim Chiok, Niraj K. Shil, Indira Mohanty, Santanu Bose

**Affiliations:** Department of Veterinary Microbiology and Pathology, College of Veterinary Medicine, Washington State University, Pullman, Washington, United States of America; University of Tennessee Health Science Center, UNITED STATES

## Abstract

Exaggerated inflammatory response results in pathogenesis of various inflammatory diseases. Tumor Necrosis Factor-alpha (TNF) is a multi-functional pro-inflammatory cytokine regulating a wide spectrum of physiological, biological, and cellular processes. TNF induces Focal Adhesion Kinase (FAK) for various activities including induction of pro-inflammatory response. The mechanism of FAK activation by TNF is unknown and the involvement of cell surface integrins in modulating TNF response has not been determined. In the current study, we have identified an oxysterol 25-hydroxycholesterol (25HC) as a soluble extracellular lipid amplifying TNF mediated innate immune pro-inflammatory response. Our results demonstrated that 25HC-integrin-FAK pathway amplifies and optimizes TNF-mediated pro-inflammatory response. 25HC generating enzyme cholesterol 25-hydroxylase (C25H) was induced by TNF via NFκB and MAPK pathways. Specifically, chromatin immunoprecipitation assay identified binding of AP-1 (Activator Protein-1) transcription factor ATF2 (Activating Transcription Factor 2) to the *C25H* promoter following TNF stimulation. Furthermore, loss of C25H, FAK and α5 integrin expression and inhibition of FAK and α5β1 integrin with inhibitor and blocking antibody, respectively, led to diminished TNF-mediated pro-inflammatory response. Thus, our studies show extracellular 25HC linking TNF pathway with integrin-FAK signaling for optimal pro-inflammatory activity and MAPK/NFκB-C25H-25HC-integrin-FAK signaling network playing an essential role to amplify TNF dependent pro-inflammatory response. Thus, we have identified 25HC as the key factor involved in FAK activation during TNF mediated response and further demonstrated a role of cell surface integrins in positively regulating TNF dependent pro-inflammatory response.

## Introduction

Exaggerated inflammation plays a critical role in pathogenesis of various inflammatory diseases like pneumonia, sepsis, diabetes, arthritis, cancer, Alzheimer’s disease [1–5]. Tumor Necrosis Factor-alpha (TNF) is a multi-functional pro-inflammatory cytokine effecting various physiological, biological, and cellular processes including immunity, inflammation, apoptosis, coagulation, endothelial cell function, insulin resistance and lipid metabolism [6–11]. TNF binds to its receptors Tumor Necrosis Factor Receptor-1 (TNFR1) and TNFR2 on the plasma membrane, which results in recruitment of multiple signal transducing adaptors that activate transcription factors AP-1 (Activator Protein-1) and NFκB [11–13]. NFκB activation by TNF plays an important role in inflammation due to production of multiple NFκB-dependent pro-inflammatory cytokines and chemokines. TNF activates focal adhesion kinase (FAK) to regulate various cellular functions of TNF including TNF mediated inflammatory response [14–23]. However, the mechanism utilized by TNF to activate FAK is unknown. Although FAK is activated by integrins, the role of integrins during TNF induced response has not yet been determined.

Recently, we identified 25-hydroxycholesterol (25HC) as a lipid ligand for integrins [24]. 25HC is an oxysterol comprising of oxygenated metabolite of cholesterol catalyzed by the enzyme cholesterol 25-hydroxylase (C25H) [25]. 25HC binding to integrins triggered FAK and NFκB activation and subsequent production of pro-inflammatory cytokines [24]. Our recent study have identified 25HC-integrin-FAK-NFκB signaling network that triggers pro-inflammatory response during activation of cytosolic pattern recognition receptor (PRR) Nod2 and infection by respiratory viruses [respiratory syncytial virus (RSV) and influenza A virus (IAV)] [24]. TNF constitutes one of the major pro-inflammatory cytokines produced following Nod2 activation and virus (RSV and IAV) infection.

Here, we investigated whether TNF can directly induce C25H leading to release of 25HC and activation of 25HC-integrin-FAK pathway for amplifying TNF-mediated pro-inflammatory response. Our studies revealed induction of C25H by TNF via MAPK (Mitogen-Activated Protein Kinase) and NFκB pathways. TNF triggered binding of ATF-2, a MAPK-dependent AP-1 transcription factor to the *C25H* promoter for transactivating C25H expression. TNF-mediated C25H induction led to 25HC production and subsequently, extracellular 25HC activated a pro-inflammatory response via 25HC-integrin-FAK pathway. Lack of C25H and blockage of integrin and FAK activation led to diminished TNF-mediated pro-inflammatory response. Thus, our study has highlighted the role of C25H-25HC-integrin-FAK signaling network in amplifying TNF-mediated pro-inflammatory response. Moreover, extracellular 25HC links the TNF-pathway to the integrin-FAK pathway to amplify TNF-dependent pro-inflammatory response.

## Materials and methods

### Mice

Female 6–8 weeks old C57BL/6J Wild type (WT) and C25H knockout (KO) mice were purchased from Jackson Laboratory (Bar harbor, ME). Animal experiments were approved and carried out in accordance with the guidelines established by the Institutional Animal Care and Use Committee (IACUC) of Washington State University.

### Cell culture

Bone marrow-derived macrophages (BMDMs) were obtained from femurs and tibia of wild type (WT) and C25H knockout (KO) mice as previously described [24]. BMDMs were cultured for 6–8 days and plated for experiments in 1640 RPMI, 10% FBS and 100 IU/ml penicillin, 100μg/ml streptomycin, and 20ng/ml GM-CSF. Human monocyte cell line (THP-1) obtained from American Type Culture Collection (ATCC) were maintained in 1640 RPMI, 10% FBS, 100 IU/ml penicillin, 100μg/ml, 1mM sodium pyruvate, 100mM HEPES, and 50μM β-mercaptoethanol. THP-1 cells were differentiated to macrophages using 100nM phorbol 12-myristate 13-acetate (PMA) for 24hrs. Mouse macrophage cell line (RAW 264.7) and mouse embryonic fibroblasts (WT and FAK KO MEFs) were obtained from ATCC and cultured in DMEM, 10% FBS, 100 IU/ml penicillin, and 100μg/ml streptomycin. Human haploid (HAP1) cells (WT and α5 integrin null cells) purchased from Horizon Discovery Inc and maintained in IMDM, 10% FBS, 100 IU/ml penicillin, 100 μg/ml streptomycin. The human epithelial cell lines 2fTGH and U5A were kindly gifted by Dr. George Stark (Cleveland Clinic, Ohio) and cultured in DMEM, 10% FBS, 100 IU/ml penicillin and 100μg/ml streptomycin.

### Antibodies and reagents

Recombinant mouse and human TNF-α were purchased from R & D systems. RPMI 1640, DMEM, IMDM, DPBS and penicillin-streptomycin were purchased from Life Technologies. Blocking antibodies; integrin α5β1 (Millipore) and Interferon Receptor 1 or IFNR1(BIO X Cell) were purchased. Isotype control antibody (control IgG) was purchased from Innovative Research. ELISA kits- 25-hydroxycholesterol (My Biosource), CCL3 (C-C Motif Chemokine Ligand 3) (Invitrogen), IL-6 (interleukin-6) (Invitrogen). Inhibitors- FAK inhibitor PF-431396 (Sigma), NF-κB inhibitor (Bay-11-7082; Invivogen), p38 inhibitor (SB203580; Invivogen), JNK (c-Jun N-terminal Kinase) inhibitor (SP600125; Invivogen) and ERK (Extracellular-signal-Regulated Kinase) inhibitor (PD98059; Invivogen). Mouse interferon-β (IFN-β) protein was purchased from Sino Biological. Phospho-FAK, FAK, phospho-IκB and IκB, STAT1 (Signal Transducer and Activator of Transcription– 1) and phospho-STAT1 antibodies were obtained from Cell Signaling Technology. C25H antibody was purchased from Santa Cruz Biotechnology and Invitrogen. The actin antibody was purchased from Bethyl Laboratories.

### TNF treatment

Cells were treated with TNF (BMDMs-100ng/ml; RAW 264.7-100ng/ml; MEFs-10ng/ml; THP-1-100ng/ml; HAP1-30ng/ml; 2fTGH and U5A-30ng/ml) for different time points as indicated in figure legends. In some experiments, RAW 264.7 cells were pre-treated with FAK inhibitor (5μM) for 1hr and subsequently treated with TNF in presence or absence of FAK inhibitor. Additionally, in some experiments BMDMs were pretreated with control IgG or α5β1 integrin blocking antibody (75μg/ml) for 2h, and subsequently treated with TNF. To assess production of IL-6, WT and FAK KO MEFs cells were treated with TNF for 16h. In some experiments, cells were pre-treated with either NFκB inhibitor [Bay-11 (10μM; 1h)] or MAPK inhibitors [p38 inhibitor (10μM; 2h), JNK inhibitor (25μM; 2h) and ERK inhibitor (25μM; 2h)]. These cells were then treated with TNF in presence and absence of inhibitors. To evaluate STAT1 activation by IFN-β in the presence and absence of IFNR1 blocking antibody, IFN-β protein was incubated with either control IgG or IFNR1 blocking antibody at 4°C for 1h. Following incubation, they were added to MEFs for 30mins, and the collected cell lysate was subjected to western blot analysis with phospho-STAT1 antibody. For IFNR1 blocking antibody experiments, cells were pre-treated with control IgG or IFNR1 antibody for 2h and subsequently treated with TNF in presence of control IgG or IFNR1 antibody.

### ELISA assay

Medium supernatants from TNF treated cells were analyzed for 25HC, CCL3 and IL-6 levels using specific ELISA kits as per manufacturer’s instructions. ELISA values of the experimental group (i.e., TNF treated cells and cells treated with TNF in the presence of FAK inhibitor or α5β1 integrin blocking antibody) represent values obtained following subtraction of background signal measured in the control group (i.e., vehicle treated cells and cells treated with TNF in the presence of vehicle treated cells or cells treated with control IgG).

### Western blot

Cells were washed with DPBS and lysed (1% Triton-X100, 1x Roche complete Mini EDTA-free protease inhibitor cocktail, 1x sodium pyrophosphate in DPBS) for 20-30mins. Cell lysates were subjected to western blot analysis. Western blot bands were visualized using ChemiDoc^™^ XRS (BioRad) and quantified using ChemiDoc^™^ XRS + software Image Lab 5.1 (BioRad).

### RealTime quantitative RT-qPCR (RT-qPCR)

Total RNA was extracted using TriZOL Reagent (Invitrogen). Isolated RNA was treated with RNase-free DNase I (Thermoscientific) and cDNA was synthesized using a High-Capacity cDNA Reverse Transcription Kit (Applied Biosystems). RT-qPCR was performed using Bio-Rad CFX Manager with 1X SSO Advanced Universal SYBR Green supermix (BioRad) using gene specific primers. Target genes were normalized to the house keeping gene GAPDH. The primers used for specific genes are as follows:

Mouse C25H forward, 5’ TGCTACAACGGTTCGGAGC 3’
Mouse C25H Reverse, 5’ AGAAGCCCACGTAAGTGATGAT 3’
Mouse GAPDH forward, 5’ CGACTTCAACAGCAACTCCCACTCTTCC 3’
Mouse GAPDH Reverse, 5’ TTGGTGGTCCAGGGTTTCTTACTCCTT 3’
Human C25H forward, 5’ GCTGGCAACGCAGTATATGA 3’
Human C25H Reverse, 5’ ACGGAAAGCCAGATGTTGAC 3’
Human GAPDH forward, 5’ GATCATCAGCAATGCCTCCT 3’
Human GAPDH reverse, 5’ TGTGGTCATGAGTCCTTCCA 3’

### Chromatin immunoprecipitation assay (ChIP-qPCR)

A putative binding motif for ATF2 was predicted within the mouse *C25H* gene promoter region using the Motif Localization Toolbox (MoLoTool) available at https://molotool.autosome.ru/ [26]. Primers against this region were designed using Primer-Blast [27]. ChIP-qPCR was performed as described previously with few modifications [28]. Mouse embryonic fibroblasts (MEFs) were treated with either vehicle or TNF (10ng/mL) for 4 hours. Formaldehyde cross-linked cells were lysed with IP buffer containing protease inhibitors (150mM NaCl, 50mM Tris-HCl pH 7.5, 5mM EDTA, NP-40 0.5%, Triton X-100 1%). Chromatins recovered from cell lysates were sheared by sonication to obtain DNA fragments between 0.5 and 1Kb. ChIP was performed with 2μg of mouse anti-ATF2 antibodies (Santa Cruz Biotechnology) or control mouse IgG (Santa Cruz Biotechnology) overnight at 4°C. ChIP reactions were immobilized on protein G-agarose (ThermoFisher Scientific). DNA was isolated using 10% Chelex100 (BioRad) followed by proteinase K treatment (Qiagen). qPCR was performed using 1X SSO Advanced Universal SYBR Green supermix (BioRad), 0.5μM forward primer (5’-GGAGCTCCGAACCGTTGTAG-3’), 0.5μM reverse primer (5’-GAAGGAGACGGAAGGGTGAC-3’) and 2μL of DNA template. Cycling conditions were set to 95°C for 5 minutes, 40 cycles at 95°C for 20 seconds, 57°C for 20 seconds and 72°C for 20 seconds, followed by melting curve analysis. Ct values were normalized by subtracting the Ct of input DNA (non-ChIP chromatins) from the Ct of each corresponding ChIP reaction. Fold enrichment was determined by 2^(CtIgG-CtATF2)^ using normalized Ct values.

### Statistical analysis

All data were analyzed using Graphpad Prism software (6.0). For ELISA, significance test was carried out using Student’s t-test. RT-qPCR data were analyzed using one-way ANOVA multiple comparison test. One-way ANOVA multiple comparison test was used for ChIP assay data analysis. Western blot densitometric values were quantified by using ChemiDoc^™^ XRS + software Image Lab 5.1 (BioRad) and Student’s t-test was utilized to determine significance.

## Results

### TNF induces C25H expression leading to production of 25HC

**Fig 1:**
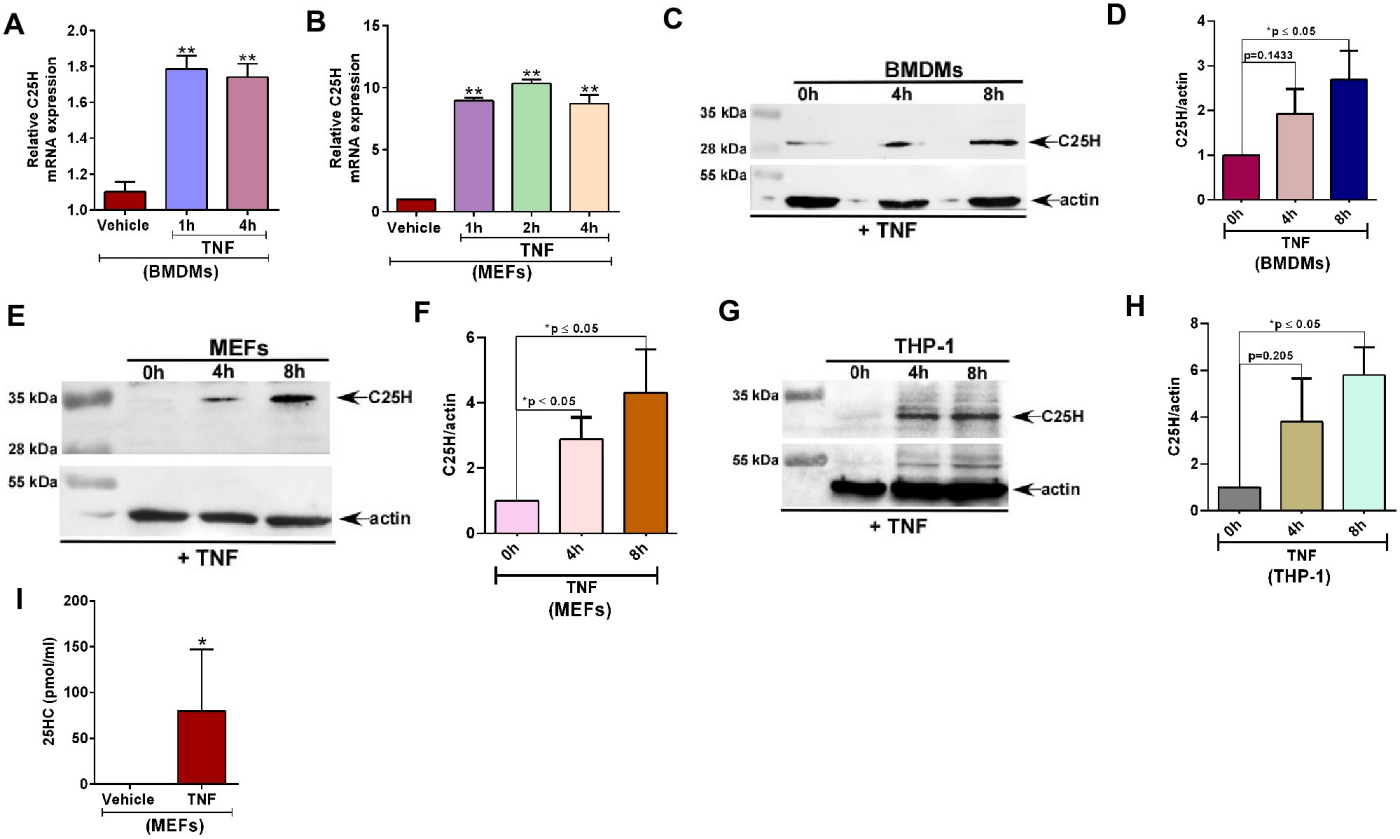
TNF induces C25H expression leading to production of 25HC. (A) BMDMs treated with TNF (100ng/ml) for 1h and 4h were analyzed for C25H mRNA expression by RT-qPCR (n = 5–6; two independent experiments). (B) MEFs treated with TNF (10ng/ml) for 1h, 2h and 4h were analyzed for C25H mRNA expression by RT-qPCR (n = 5–6; two independent experiments). (C) BMDMs treated with TNF (100ng/ml) for 4h and 8h were analyzed for C25H protein expression by western blotting. (D) Densitometry analysis of C25H protein levels relative to actin (C25H/actin) in BMDMs treated with TNF for 0h, 4h and 8h. C25H/actin ratio levels were normalized to 0h treatment. (E) MEFs treated with TNF (10ng/ml) for 4h and 8h were analyzed for C25H protein expression by western blotting. (F) Densitometry analysis of C25H protein levels relative to actin (C25H/actin) in MEFs treated with TNF for 0h, 4h and 8h. C25H/actin ratio levels were normalized to 0h treatment. (G) Human macrophage THP-1 cells treated with TNF (100ng/ml) for 4h and 8h were analyzed for C25H protein expression by western blotting. (H) Densitometry analysis of C25H protein levels relative to actin (C25H/actin) in THP-1 cells treated with TNF for 0h, 4h and 8h. C25H/actin ratio levels were normalized to 0h treatment. (I) 25HC production from MEFs treated with TNF (10ng/ml) for 4h were analyzed by ELISA (n = 9; three independent experiments). ELISA data are shown as Mean ± SEM. *p ≤ 0.05 using a Student’s t-test. RT-qPCR data are shown as Mean ± SEM. **p ≤ 0.0001 using one-way ANOVA multiple comparison test. Immunoblot images are representative from three-four independent experiments. The densitometric values represent the mean ± SEM from three- four independent experiments. *p < 0.05 determined by Student’s t-test.

Previous studies have shown that C25H is an interferon (IFN) inducible gene [29–31]. *C25H* gene is induced by both type-I (IFN-β) and type-II (IFN-γ) interferons [29–31]. We investigated whether another cytokine like TNF can also regulate C25H expression. For this study, we treated macrophages (mouse bone marrow derived macrophages or BMDMs) with TNF followed by real time quantitative RT-qPCR (RT-qPCR) analysis to detect C25H mRNA expression. C25H induction was noted within 1h of TNF treatment and was maintained even at 4h post-TNF treatment (Fig 1A). This phenomenon was not limited to myeloid cells like macrophages, since TNF also induced C25H expression in non-myeloid mouse embryonic fibroblast (MEFs) cells (Fig 1B). In accord with the PCR results, C25H protein expression was also induced by TNF in BMDMs and MEFs as deduced by western blot analysis with anti-C25H antibody (Fig 1C–1F). C25H protein induction by TNF was not limited to murine cells since we observed TNF-mediated C25H protein expression induction in the human macrophage THP-1 cell-line (Fig 1G and 1H). Noteworthy, molecular weight of C25H is specific to each cell type depending on its glycosylation status in a particular cell [25]. Accordingly, we observed variation in the molecular weight of C25H protein in the three cell-lines (THP-1, MEFs and BMDMs) used in our study (Fig 1C, 1E and 1G).

C25H is required for generating 25HC. Therefore, we evaluated 25HC levels in TNF treated cells. Expression of C25H by TNF was functional, since we observed production of 25HC from TNF treated MEFs (Fig 1I) and macrophages (S1A Fig). We noted production of 80 pmol/ml of 25HC from MEFs following 4h TNF treatment (Fig 1I). However, treatment of MEFs with 80 pmol/ml of 25HC for 4h did not induce C25H expression (S1B Fig). This result suggested that TNF directly induces C25H expression during early treatment time-periods in the absence of autocrine/paracrine action of 25HC. These studies have identified C25H as a TNF-inducible gene involved in 25HC release from TNF treated cells.

### 25HC promotes optimal TNF mediated pro-inflammatory response and NFκB activation

**Fig 2:**
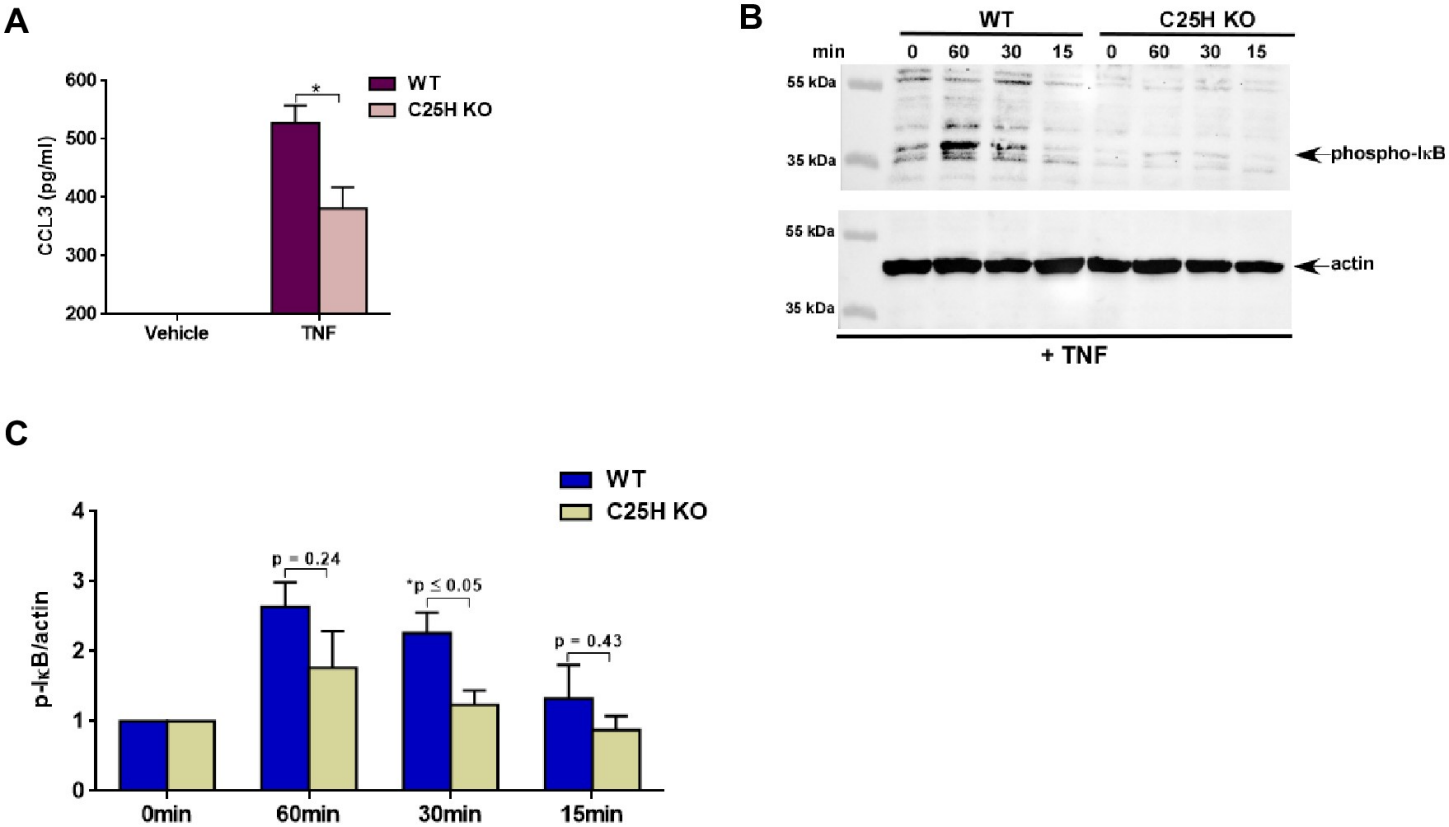
25HC is required for optimal TNF mediated pro-inflammatory response and NFκB activation. (A) Medium supernatant collected from WT and C25H KO BMDMs treated with TNF (100ng/ml) for 16h was analyzed for CCL3 production by ELISA (n = 12; three independent experiments). (B) Cell lysate collected from WT and C25H KO cells treated with TNF (100ng/ml) for 0–60 minutes were subjected to western blot analysis to detect status of phosphorylated IκB (phospho-IκB). The immunoblot is representative of three independent experiments with similar results. (C) Densitometric analysis of the western blot presented in Fig 2b. The densitometric quantification values for phospho-IκB immunoblot represent the ratio of phospho-IκB:actin and the fold-induction was calculated after normalizing with the control 0 min group. ELISA data are shown as Mean ± SEM. *p ≤ 0.05 using a Student’s t-test. The densitometric values represent the mean ± SEM from three independent experiments. The p value shown in the figure was calculated using a Student’s t-test (*p ≤ 0.05).

Since TNF induced C25H expression and concomitantly released 25HC (Fig 1), we next examined the functional role of 25HC during TNF-mediated response. For these studies, we used C25H knockout (KO) bone marrow derived macrophages (BMDMs) as these cells do not produce 25HC due to lack of C25H. To investigate the role of 25HC during TNF-dependent pro-inflammatory response, we treated wild type (WT) and C25H KO cells with TNF. Production of TNF-dependent pro-inflammatory chemokine CCL3 from WT and C25H KO BMDMs was assessed by ELISA analysis. Significant reduction in CCL3 production was noted following TNF treatment of C25H KO BMDMs (Fig 2A) indicating that 25HC plays a critical role during TNF-dependent response.

One of the hallmarks of TNF signaling is activation of the transcription factor NFκB which is involved in expression of various pro-inflammatory cytokines and chemokines [11]. Therefore, we next examined whether 25HC contributes to NFκB activation during TNF signaling. WT and C25H KO cells treated with TNF were subjected to western blot analysis with antibody specific for phosphorylated IκB. We observed loss of NFκB activation (as shown by reduced levels of phosphorylated-IκB) in C25H deficient cells treated with TNF (Fig 2B). Densitometric analyses of the western blot data revealed drastic reduction of NFκB activity (i.e., reduced phospho-IκB levels) following TNF treatment of C25H KO cells (Fig 2C). These results highlighted a role of 25HC in magnifying TNF-mediated response.

### FAK activation by 25HC is required for optimal TNF-mediated pro-inflammatory response and NFκB activation

FAK is a critical adaptor protein in integrin signaling pathway [32, 33]. TNF activates FAK in multiple cells including epithelial cells, endothelial cells, fibroblasts, and muscle cells [14–23] and such activation is required for optimal production of pro-inflammatory cytokines IL-6 and IL-8 [19–21]. Although FAK plays an important role during TNF-mediated response, the mechanism of FAK activation by TNF is still unknown. We recently showed that 25HC activates FAK in macrophages and MEFs following Nod2 activation and virus infection [24]. Since TNF also triggered 25HC production in immune cells (i.e., macrophages) (Fig 1C), we speculated that TNF may also activate FAK in macrophages via 25HC mediated integrin activation.

**Fig 3:**
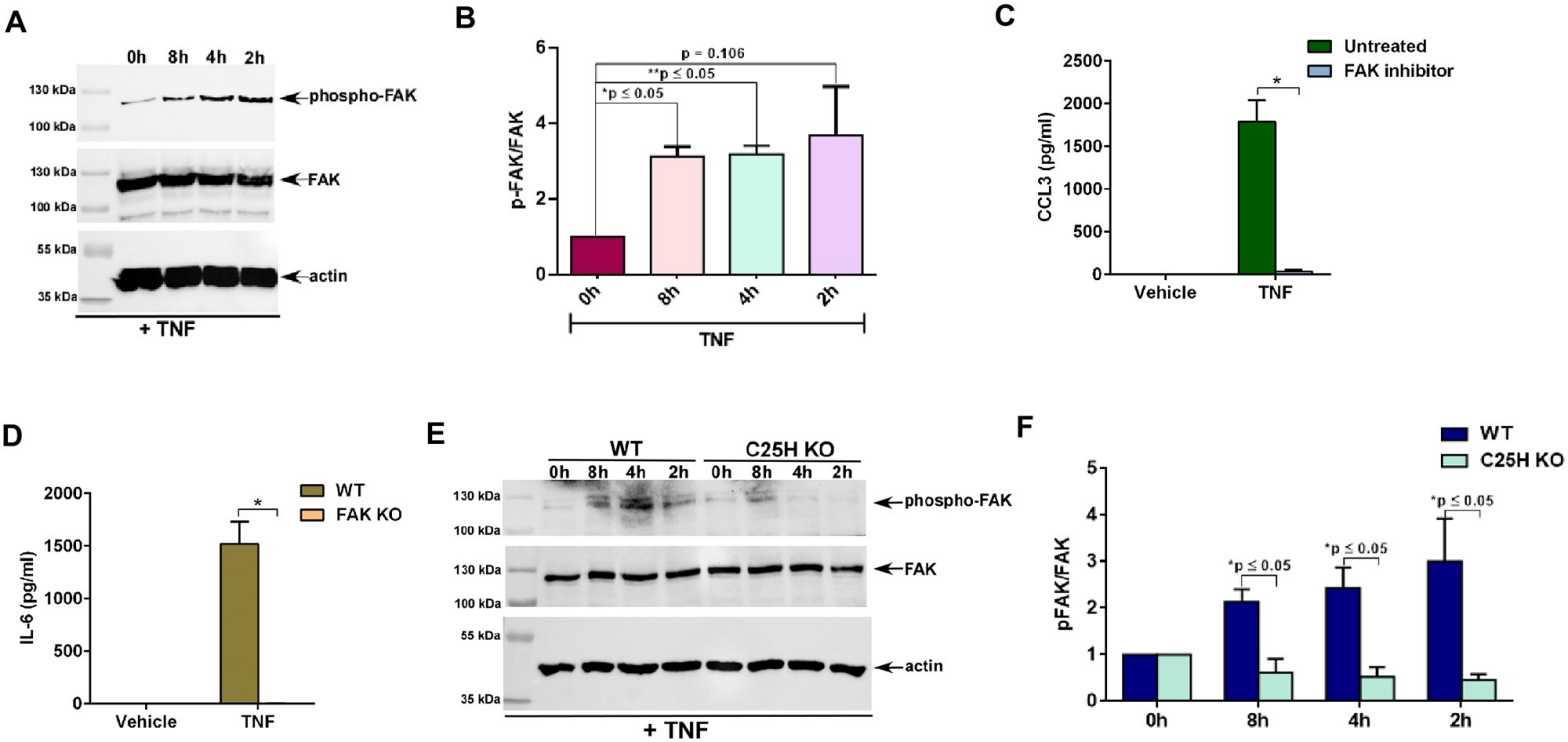
25HC produced by TNF activates FAK and FAK activation is required for optimal TNF-mediated pro-inflammatory response and NFκB activation. (A) Cell lysate from THP-1 macrophages treated with 100ng/ml of TNF for the indicated time points were subjected to western blot to detect status of phosphorylated FAK (phospho-FAK). The immunoblot is representative of three independent experiments with similar results. (B) Densitometric analysis of the western blot presented in Fig 3a. The densitometric quantification values for phospho-FAK immunoblot represent the ratio of phospho-FAK:FAK and the fold-induction was calculated after normalizing with the control 0h group. (C) Medium supernatant collected from RAW264.7 macrophages treated with TNF for 8h in the presence or absence (untreated vehicle control) of FAK inhibitor (5μM) was analyzed for CCL3 production by ELISA (n = 12; three independent experiments). (D) Medium supernatant collected from WT and FAK KO MEFs treated with TNF (10ng/ml;16h) was analyzed for IL-6 production by ELISA (n = 9; three independent experiments). (E) Cell lysate from WT and C25H KO BMDMs treated with TNF (100ng/ml) for the indicated time points were subjected to western blot to detect status of phosphorylated FAK (phospho-FAK). The immunoblot is representative of four independent experiments with similar results. (F) Densitometric analysis of the western blot presented in Fig 3e. The densitometric quantification values for phospho-FAK immunoblot represent the ratio of phospho-FAK:FAK and the fold-induction was calculated after normalizing with the control 0h group. ELISA data are shown as Mean ± SEM. *p ≤ 0.05 using a Student’s t-test. The densitometric values represent the mean ± SEM from four independent experiments. The p value shown in the figure was calculated using a Student’s t-test (*p and **p ≤ 0.05).

Tyrosine residues of FAK are phosphorylated following integrin activation and therefore, phosphorylated form of FAK represents the activated form of FAK [32, 33]. To examine FAK activation status, cell lysates derived from TNF treated macrophages were subjected to western blot analysis with antibody specific for phospho-FAK. TNF triggered FAK activation in macrophages since we detected phosphorylated FAK in TNF treated macrophages (Fig 3A). Densitometric analyses of the western blot result revealed robust FAK induction in macrophages following TNF treatment (Fig 3B). We next investigated whether FAK activation plays a role in TNF-mediated induction of the pro-inflammatory response by assaying production of the pro-inflammatory chemokine CCL3 from cells either lacking FAK expression or treated with FAK inhibitor. TNF-induced production of CCL3 was abrogated in macrophages treated with FAK inhibitor (Fig 3C). Accordingly, treatment of WT and FAK knockout (KO) MEFs with TNF revealed drastic loss of IL-6 production from FAK KO cells (Fig 3D). Thus, our studies have demonstrated a role of FAK in positively regulating TNF-mediated pro-inflammatory response in macrophages.

Finally, the role of 25HC in inducing FAK signaling during TNF-mediated pro-inflammatory response was investigated by evaluating FAK activation status in WT and C25H KO macrophages treated with TNF. While TNF triggered FAK activation in WT cells, as indicated by its phosphorylation, such activation was lacking in C25H KO cells (Fig 3E). Densitometric analyses of the western blot data revealed loss of FAK activation (i.e., reduced phospho-FAK levels) following TNF treatment of C25H KO macrophages (Fig 3F). Our study revealed that during TNF mediated response, 25HC promotes activation of FAK signaling, which is required to amplify TNF-dependent pro-inflammatory response.

### α5 integrin contributes to optimal TNF-mediated pro-inflammatory response and NFκB activation

We recently identified α5β1 integrin complex involved in triggering 25HC-mediated pro-inflammatory response during Nod2 activation and virus infection [24]. Since TNF activated FAK (Fig 3A and 3B) and induced 25HC production (Fig 1C), we next evaluated the role of α5 integrin in regulating TNF-mediated response.

**Fig 4:**
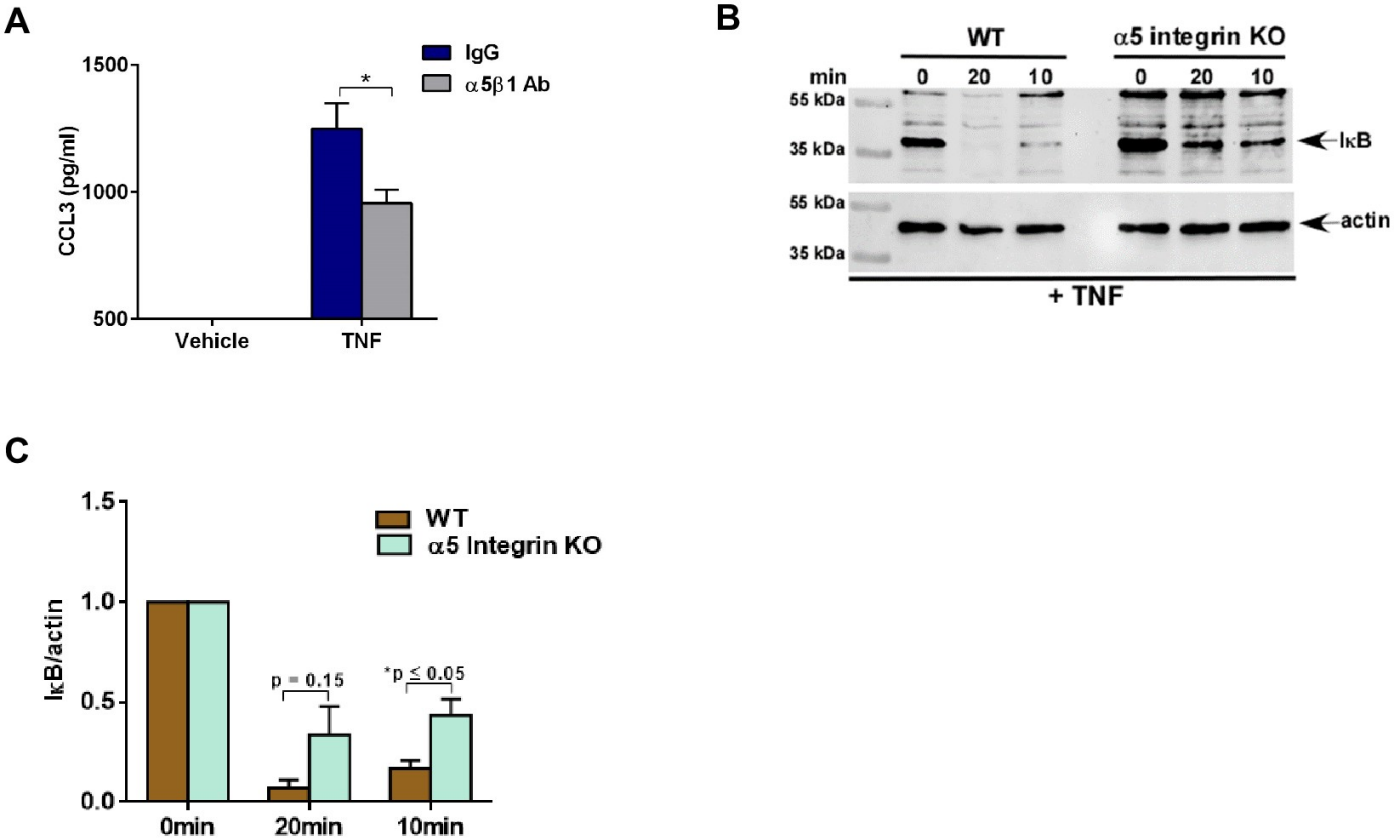
α5 integrin is required for optimal TNF-mediated pro-inflammatory response and NFκB activation. (A) Medium supernatant collected from BMDMs treated with TNF (100ng/ml) for 16h in the presence of either control IgG or α5β1 blocking antibody (75μg/ml) was analyzed for CCL3 production by ELISA (n = 8; two independent experiments). (B) Cell lysate collected from WT and α5 integrin KO HAP-1 cells treated with TNF (30ng/ml) for indicated time periods were subjected to western blot analysis to detect status of IκB protein. The immunoblot is representative of three independent experiments with similar results. (C) Densitometric analysis of the western blot presented in Fig 4b. The densitometric quantification values for IκB immunoblot represent the ratio of IκB:actin and the fold-induction was calculated after normalizing with the control 0 min group. ELISA data are shown as Mean ± SEM. *p ≤ 0.05 using a Student’s t-test. The densitometric values represent the mean ± SEM from three independent experiments. The p value shown in the figure was calculated using a Student’s t-test (*p ≤ 0.05).

We first used α5β1 integrin blocking antibody to assess the role of integrin during TNF signaling. Inhibition of cell surface α5β1 integrin by integrin blocking antibody in TNF treated macrophages significantly reduced CCL3 production compared to IgG-treated control cells (Fig 4A). Thus, integrins such as α5β1 integrin may play a role in amplifying TNF-mediated pro-inflammatory response.

The above results suggested that lack of α5 integrin expression may also lead to loss of NFκB activation by TNF. To examine this scenario, we utilized α5 integrin knockout (α5 KO) human haploid (HAP1) cells [24, 34]. These cells generated by CRISPR-Cas9 technology lack expression of α5 integrin and have been used previously to study the role of α5 integrin during 25HC mediated signaling pathway [24]. NFκB activation status in α5 KO and WT cells treated with TNF was assessed by western blot analysis using IκB antibody. We observed reduced NFκB activation in TNF treated α5 KO cells compared to WT cells (Fig 4B). Densitometric analyses of the western blot data revealed significantly diminished NFκB activity (i.e., reduced IκB protein degradation) following treatment of α5 KO cells (Fig 4C). These results demonstrated that integrins like α5β1 integrin play a role in conferring optimal pro-inflammatory response by TNF. This is the first report demonstrating a role of integrins in regulating TNF-dependent response.

### TNF induces C25H expression via type-I interferon independent mechanism

Enzymatic activity of C25H is required for generation of 25HC [24, 25, 29–31, 35, 36]. Various stimuli (e.g., virus infection, activation of pattern recognition receptors like Nod2, TLR) induce C25H gene expression leading to production of 25HC [24, 25, 29–31, 35, 36]. Since TNF induced C25H (Fig 1A and 1B) and released 25HC (Fig 1C), we investigated the mechanism by which TNF regulates C25H gene expression. Previous studies have shown that C25H is an interferon (IFN) inducible gene since both IFN-β (type-I IFN) and IFN-γ (type-II IFN) induced C25H mRNA expression [29–31]. In addition, direct binding of IFN-γ activated STAT-1 transcription factor to C25H promoter has been observed in macrophages [30]. Therefore, we investigated whether TNF utilizes the interferon pathway to induce C25H.

**Fig 5:**
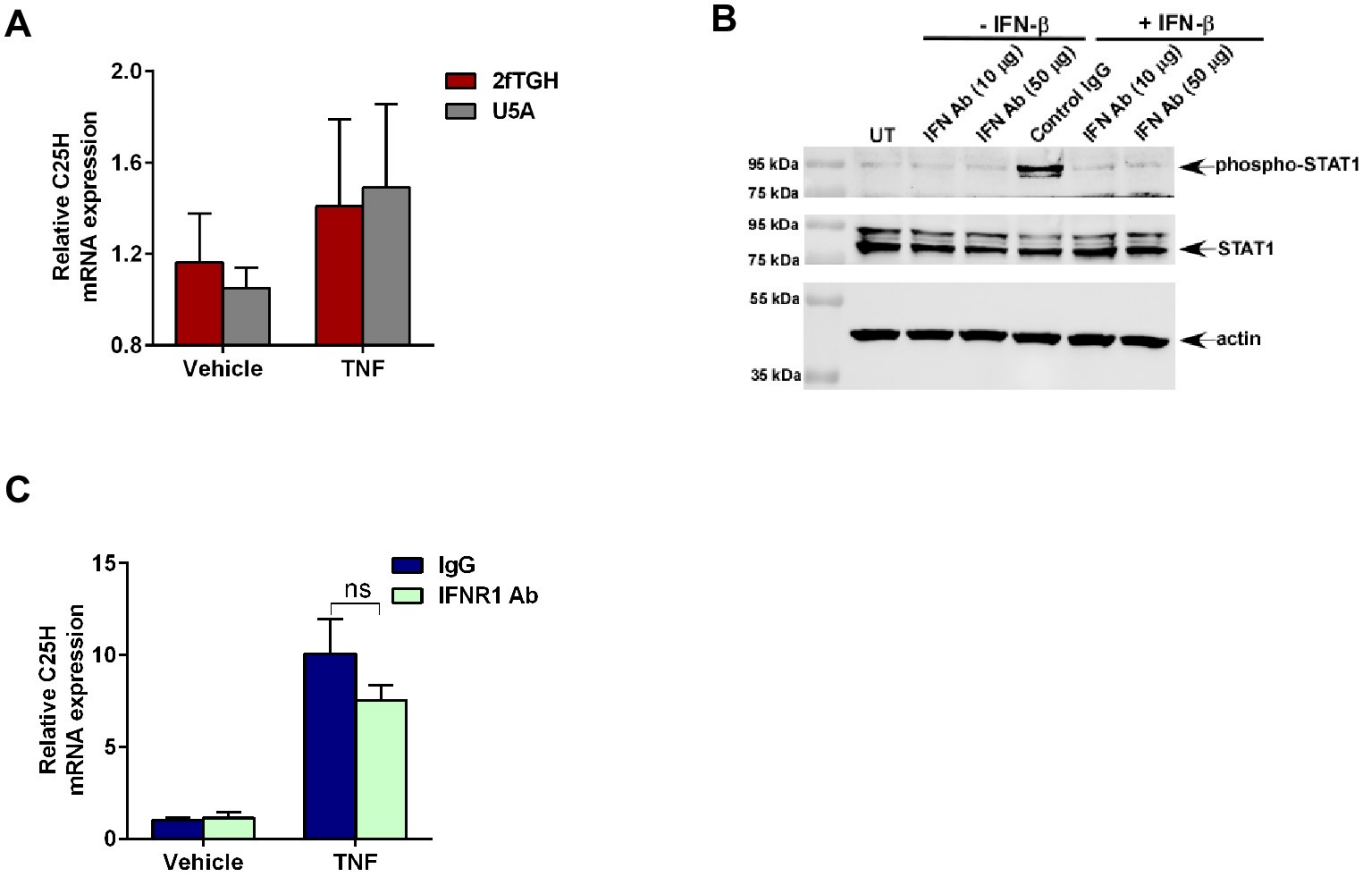
TNF induces C25H expression via type-I interferon independent mechanism. (A) 2fTGH and U5A cells treated with TNF (30ng/ml) for 1h were analyzed for C25H mRNA expression by RT-qPCR (n = 5–6; two independent experiments). (B) Cell lysate collected from MEFs treated with IFN-β protein (100 IU/ml) for 30mins in the presence of either control IgG or IFNR1 blocking antibody (IFN Ab) (50μg/ml) were subjected to western blot analysis to detect status of phosphorylated STAT-1 (phospho-STAT1) protein. The immunoblot is representative of two independent experiments with similar results. (C) MEFs treated with TNF (10ng/ml) for 4h in the presence of either control IgG or IFN Ab (50μg/ml) were analyzed for C25H mRNA expression by RT-qPCR (n = 5–6; two independent experiments). RT-qPCR data are shown as Mean ± SEM by using one-way ANOVA multiple comparison test. ns; not significant.

For these studies we first utilized epithelial cells deficient in type-I interferon signaling. We used type-I interferon receptor (IFNAR2) lacking U5A cells and the parental wild-type 2fTGH cells. 2fTGH and U5A cells treated with TNF were subjected to RT-qPCR analysis to detect expression of C25H. Surprisingly, lack of type-I interferon signaling did not affect C25H expression following TNF treatment (Fig 5A). Type-I interferon signaling deficient U5A cells and parental 2fTGH cells exhibited similar C25H expression levels following TNF treatment (Fig 5A). Additionally, type-II interferon was not involved in TNF-mediated C25H gene expression since epithelial cells (i.e., 2fTGH and U5A cells) do not produce IFN-γ.

We validated this result by using IFNAR blocking antibody. We selected MEFs for studies related to TNF-mediated C25H expression due to their robust expression of C25H following TNF treatment (Fig 1B). The efficiency of IFNAR blocking antibody in MEFs was borne out by the observation that STAT-1 activation (as analyzed by detecting phosphorylated form of STAT-1) was drastically reduced in cells treated with IFN-β in the presence of the blocking antibody (Fig 5B). To evaluate the role of interferon pathway, C25H expression was analyzed by RT-qPCR in MEFs treated with TNF in the presence of the IFNAR blocking antibody. Type-I interferon is not involved in *C25H* gene induction following TNF treatment since there was no significant difference in C25H expression in cells treated with control IgG versus IFNAR blocking antibody (Fig 5C). Furthermore, type-II interferon was not involved during TNF-mediated *C25H* gene expression since fibroblasts (i.e., MEFs) do not produce IFN-γ. Therefore, our results demonstrated interferon independent induction of C25H expression by TNF.

### MAPK and NFκB pathways are required for TNF-mediated C25H expression

One study suggested that C25H mRNA expression can be modulated by TLR4 via NFκB and MAPK-dependent JNK pathways [35]. To date, it is unknown whether NFκB and MAPK regulate C25H expression via direct binding of NFκB and/or AP-1 (AP-1 transcription factors are activated by MAPK pathway) to the C25H promoter or if an indirect mechanism is involved.

**Fig 6:**
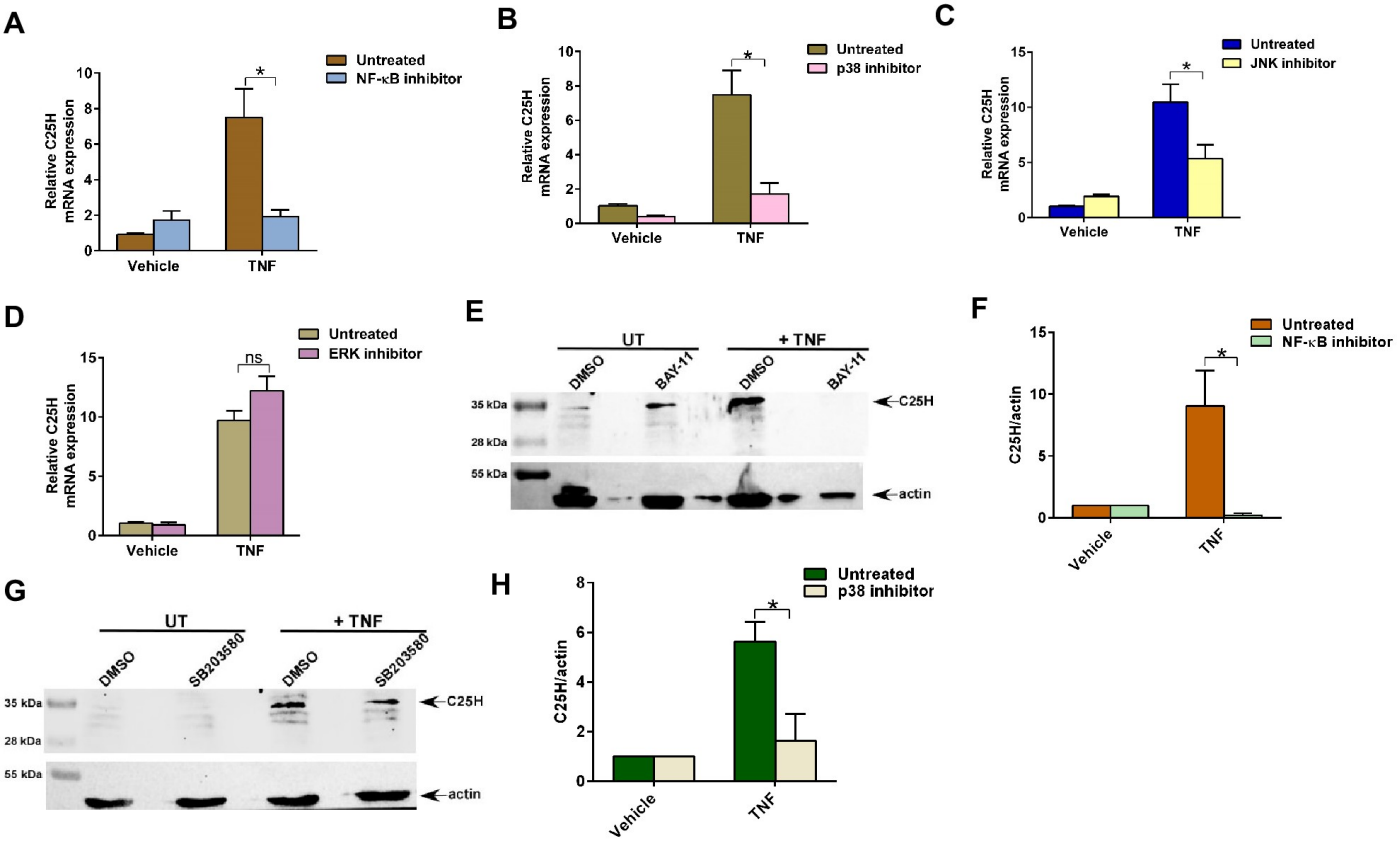
MAPK and NFκB pathways are required for TNF-mediated C25H expression. (A) MEFs treated with TNF (10ng/ml) for 4h in the absence or presence of NFκB inhibitor Bay-11 (10μM) were analyzed for C25H mRNA expression by RT-qPCR (n = 5–6; two independent experiments). (B) MEFs treated with TNF (10ng/ml) for 4h in the absence or presence of p38 inhibitor SB203580 (10μM) were analyzed for C25H mRNA expression by RT-qPCR (n = 5–6; two independent experiments). (C) MEFs treated with TNF (10ng/ml) for 4h in the absence or presence of JNK inhibitor SP600125 (25μM) were analyzed for C25H mRNA expression by RT-qPCR (n = 5–6; two independent experiments). (D) MEFs treated with TNF (10ng/ml) for 4h in the absence or presence of ERK inhibitor PD98059 (25μM) were analyzed for C25H mRNA expression by RT-qPCR (n = 5–6; two independent experiments). (E) MEFs treated with TNF (10ng/ml) for 8h in the absence or presence of NFκB inhibitor Bay-11 (10μM) were analyzed for C25H protein expression by western blotting. (F) Densitometry analysis of C25H protein levels relative to actin (C25H/actin) in MEFs treated with TNF for 8h in the absence or presence of NFκB inhibitor Bay-11 (10μM). C25H/actin ratio levels were normalized to vehicle treated cells. G) MEFs treated with TNF (10ng/ml) for 8h in the absence or presence of p38 inhibitor SB203580 (10μM) were analyzed for C25H protein expression by western blotting. (H) Densitometry analysis of C25H protein levels relative to actin (C25H/actin) in MEFs treated with TNF for 8h in the absence or presence of p38 inhibitor SB203580 (10μM). C25H/actin ratio levels were normalized to vehicle treated cells. RT-qPCR data are shown as Mean ± SEM by using one-way ANOVA multiple comparison test. *p ≤ 0.05. ns; not significant. Immunoblot images are representative from three independent experiments. The densitometric values represent the mean ± SEM from three independent experiments. *p < 0.05 determined by Student’s t-test.

We first evaluated the role of NFκB in TNF-mediated C25H expression by treating MEFs with TNF in the presence of the NFκB inhibitor BAY-11. Blocking the NFκB pathway in TNF treated cells resulted in significant loss of C25H expression (Fig 6A). This result demonstrated TNF-mediated activation of NFκB play an essential role in *C25H* gene expression.

MAPK signaling pathway regulates various cellular activities in response to diverse stimuli [37]. The three MAPK pathways identified in mammals are ERK, JNK and p38 pathways. One study reported that blocking the JNK pathway with JNK inhibitor reduced C25H expression following TLR4 activation [35]. However, it is still unknown whether C25H is a bona fide MAPK-inducible gene since direct binding of MAPK-dependent AP-1 transcription factor(s) to the *C25H* promoter has not been documented. Interestingly, TNF is known to activate the MAPK pathway in various cells to modulate wide spectrum of cellular and biological activities [38–51]. However, detailed mechanism by which MAPK pathway contributes to TNF-mediated pro-inflammatory response is lacking.

To study the role of MAPK pathway, we treated TNF-primed MEFs with inhibitors specific for ERK, JNK and p38 pathways. Analysis of C25H expression by RT-qPCR revealed significant reduction in C25H expression following inhibition of p38 and JNK pathways (Fig 6B and 6C). The requirement of p38 and JNK pathways for C25H expression was specific since blocking ERK pathway in TNF treated cells did not result in loss of C25H expression (Fig 6D). Loss of C25H mRNA expression with the NFκB and MAPK inhibitors also led to significant reduction in C25H protein expression. Treatment of cells with NFκB inhibitor BAY-11 resulted in drastic loss of C25H protein expression following TNF treatment as analyzed by western blotting of cell lysates with anti-C25H antibody (Fig 6E and 6F). Enhanced exposure of the blot indicated complete abrogation of TNF-mediated C25H protein expression in BAY-11 treated cells (S2 Fig). In addition, western blot analysis with anti-C25H antibody revealed significant loss of TNF-dependent C25H protein expression following inhibition of p38 MAPK signaling with SB203580 (Fig 6G and 6H). Thus, we have identified *C25H* as a MAPK inducible gene and showed a role of p38 and JNK in inducing C25H expression in TNF treated cells. Additionally, our study suggests that while NFκB is absolutely required for C25H expression during TNF signaling, MAPK signaling plays an important role in facilitating optimal C25H expression during this event.

### TNF promotes binding of AP-1 transcription factor ATF2 to the C25H promoter

**Fig 7:**
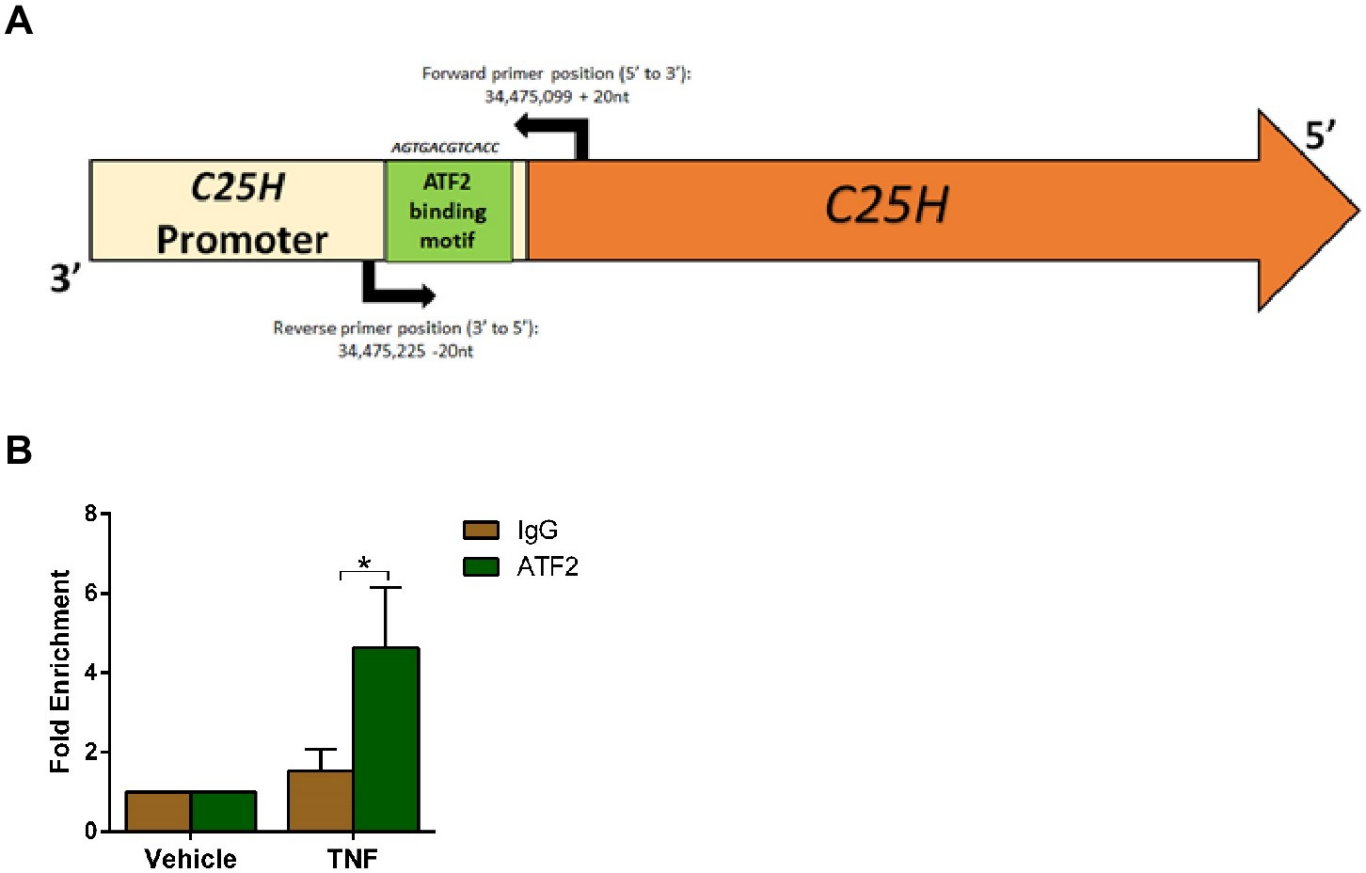
Direct binding of ATF2 transcription factor to the *C25H* promoter following activation of TNF signaling. (A) A schematic diagram of the mouse *C25H* promoter (light yellow) and mouse *C25H* gene (orange) is shown. ATF2 binding region is shown in green along with the ATF2 binding sequence in the *C25H* promoter. The arrows with numbers represent the position of the forward and reverse primers used for the ChIP-qPCR assay. (B) Chromatin was isolated from MEFs treated with TNF (10ng/ml) for 4h. ChIP-qPCR assay was performed by using ATF2 antibody, control IgG and primers to amplify the genomic region flanking the ATF2 binding site in the *C25H* promoter. Location of the primers is shown in Fig 7a. ChIP-qPCR data is shown as Mean ± SEM from three independent experiments (n = 3). *p ≤ 0.05 using one-way ANOVA multiple comparison test.

Although STAT1 and NFκB transcriptional factors have been implicated in transactivation of C25H gene [29–31, 35], the role of MAPK dependent AP-1 transcription factor(s) in C25H transactivation has not been assessed. Loss of TNF-mediated C25H induction following inhibition of JNK and p38 pathways (Fig 6B and 6C) suggested that transcription factors related to these pathways may transactivate C25H gene via direct binding to its promoter. We utilized bioinformatic tools to screen for potential AP-1 transcription factor binding sites in the C25H promoter region. We identified a potential binding site for the transcription factor ATF2 (activated by the JNK and p38 pathways) in the promoter region of *C25H* gene (Fig 7A). This ATF2 binding site (AGTGACGTCACC) in the C25H promoter region contains the consensus sequence identified for AP-1 (5’-TGAC/GTCA-3’) [52, 53]. This ATF2 binding site (*p* = 6.18x10^-6^) is located at 73 nt from the translational initiation codon within *C25H* gene and is conserved in both human and mouse *C25H* promoter regions.

To evaluate direct binding of ATF2 to the *C25H* promoter following TNF treatment, we performed chromatin immunoprecipitation (ChIP) assay followed by real time quantitative PCR (ChIP-qPCR). ChIP-qPCR analysis revealed recruitment of ATF2 to the *C25H* promoter following activation of TNF signaling (Fig 7B). We observed 3-7fold enrichment for ATF2 binding to the *C25H* promoter (Fig 7B). This level of fold enrichment is typical for ATF2 binding to promoters as previously reported [54–57]. These studies have identified AP-1 (i.e., ATF2) as a C25H transcription factor. In addition, we showed that TNF triggers direct binding of ATF2 to the *C25H* promoter for transactivation of *C25H* gene.

## Discussion

Tumor Necrosis Factor-alpha (TNF) is a multi-functional pro-inflammatory cytokine that regulates wide spectrum of physiological, biological and cellular processes including immunity, inflammation, apoptosis, coagulation, endothelial cell function, insulin resistance and lipid metabolism [6–23]. Thus, regulating TNF response is critical to maintain cellular and tissue homeostasis. Particularly, TNF is a potent pro-inflammatory cytokine involved in inducing inflammation. While inflammation is necessary for certain immune-pathological responses like host defense to clear pathogens, exaggerated inflammatory response is detrimental and can therefore manifest as inflammatory diseases like pneumonia, sepsis, diabetes, arthritis, cancer, and Alzheimer’s disease [1–5].

TNF binds to its cognate cell surface receptors, TNFR1 and TNFR2 to activate a signal transduction cascade that culminates in activation of MAPK and NFκB pathways [11–13]. These pathways, via AP-1 and NFκB transcription factors, are involved in transcriptional activation of genes involved in inflammation, apoptosis, and cell migration among others. Apart from these pathways, numerous studies have reported activation of FAK pathway by TNF which contributes to various cellular functions including TNF mediated inflammatory response [14–23]. Although TNF activates FAK in multiple cells including epithelial cells, endothelial cells, fibroblasts, muscle cells, the mechanism by which TNF activates FAK was unknown. In addition, a previous study showed that FAK is required for TNF mediated IL-6 production by an unidentified mechanism [58]. We recently identified the lipid oxysterol 25HC as an extracellular soluble factor amplifying pro-inflammatory response following Nod2 activation and respiratory virus infection [24]. We showed that 25HC directly binds to integrins to activate integrin-FAK pathway for triggering pro-inflammatory response. In the current study, we showed the involvement of 25HC and integrin in relaying FAK-dependent pro-inflammatory response following stimulation of cells with TNF. Our studies have revealed that TNF activates FAK via production of 25HC, which interacts with cell surface integrin to promote activation of FAK signaling. Thus, 25HC-integrin-FAK pathway amplifies TNF-mediated pro-inflammatory response.

Our studies have highlighted a previously unknown mechanism associated with TNF signaling. We report that 25HC-integrin-FAK signaling is an essential component of TNF-mediated pro-inflammatory response. Thus, our studies suggest MAPK/NFκB-C25H-25HC-integrin-FAK signaling network playing an important role in amplifying TNF-mediated pro-inflammatory response and during this event 25HC acts as a “bridge” to link TNF pathway to integrin-FAK pathway for pro-inflammatory response amplification.

Recent studies have demonstrated a role of 25HC in modulating innate immunity. 25HC regulates immune response mediated by type-I and type-II interferons [29–31]. Furthermore, 25HC is involved in IL-1β production by inflammasome and TLR dependent pro-inflammatory response [30, 35, 36, 59, 60]. We recently demonstrated that 25HC also participates plays a key role in amplifying Nod2 dependent pro-inflammatory response [24]. Several studies have also implicated 25HC in antiviral defense by inhibiting virus entry and post-entry steps of enveloped viruses [29, 30]. In the current study, we have identified 25HC as an important component of functional signaling transduction pathway induced by TNF, an important cytokine controlling various biological and cellular functions.

C25H has been reported to be a type-I (IFN-β) and type-II (IFN-γ) interferon inducible gene [29–31]. STAT-1 activation by IFN-γ results in binding of STAT-1 to the *C25H* promoter and resulting expression of *C25H* gene [30]. Additionally, one study also suggested that NFκB and MAPK (JNK pathway) may also be involved in C25H gene transactivation following TLR4 activation [35]. This study showed that NFκB and JNK inhibitors reduced C25H mRNA levels following TLR4 activation [35]. However, whether C25H gene expression was induced due to direct binding of NFκB and/or AP-1 factor (AP-1 transcription factors are activated by MAPK pathway) to the C25H promoter or if it occurred via an indirect mechanism was not determined. Another recent study observed C25H induction following Zika virus infection via type-I interferon independent STAT1-dependent pathway [61]. This study also noted induction of C25H by TNF in THP-1 macrophages via STAT-1 since JAK inhibitor 1 reduced TNF-mediated C25H induction by 40% [61], thus suggesting additional STAT1 independent pathway(s) playing a role in TNF mediated C25H induction. Nevertheless, C25H is considered as an interferon inducible and STAT1 dependent gene based on the stimulus (e.g., LPS, virus). However, we have now demonstrated an involvement of MAPK (AP-1) pathway in transactivating C25H expression by TNF. We show that MAPK pathways (JNK and p38 pathways) activated by TNF are required for *C25H* gene expression. More importantly, we showed direct binding of AP-1 transcription factor ATF2 to the *C25H* promoter.

In summary, our studies have identified MAPK/NFκB-C25H-25HC-integrin-FAK signaling network as an important regulator of TNF-mediated innate immune response.

## Supporting information

S1 Fig (A) 25HC production from RAW264.7 macrophages treated with TNF (100ng/ml) for 8h were analyzed by ELISA (n = 9; three independent experiments). (B) MEFs treated with either 25HC (80 pmol/ml) or TNF (10ng/ml) for 4h were analyzed for C25H mRNA expression by RT-qPCR (n = 12; four independent experiments). ELISA data are shown as Mean ± SEM. *p ≤ 0.05 using a Student’s t-test. RT-qPCR data are shown as Mean ± SEM.*p ≤ 0.05 using one-way ANOVA multiple comparison test.
(PDF)

S2 Fig Higher exposure of the C25H blot shown in Fig 6E. (PDF)

S1 Raw images Uncropped blot images. (PDF)

## Abbreviations

25HC: 25-hydroxycholesterol
ATF2: Activating Transcription Factor 2
BMDMs: bone marrow derived macrophages
C25H: cholesterol 25-hydroxylase
CCL3: C-C Motif Chemokine Ligand 3
ChIP: chromatin immunoprecipitation
ERK: extracellular signal-regulated kinase
FAK: focal adhesion kinase
IFN: interferon
IL-6: interleukin-6
JNK: c-Jun N-terminal kinase
MAPK: Mitogen-activated protein kinase
MEFs: mouse embryo fibroblasts
PMA: phorbol 12-myristate 13-acetate
TNF: tumor necrosis factor-alpha
TNFR: tumor necrosis factor receptor
WT: wild type
