# Supplementary figures and images for: Tumor Necrosis Factor-alpha utilizes MAPK/NFκB pathways to induce cholesterol-25 hydroxylase for amplifying pro-inflammatory response via 25-hydroxycholesterol-integrin-FAK pathway
Source: PLoS One. 2021 Sep 22;16(9):e0257576. doi: 10.1371/journal.pone.0257576 (PMC8457477; doi:10.1371/journal.pone.0257576)

# S1 Figure

**A**

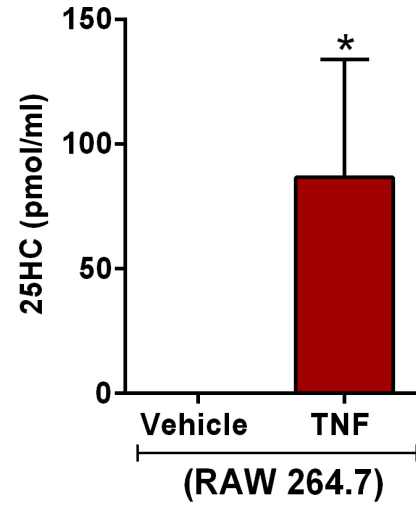

**B**

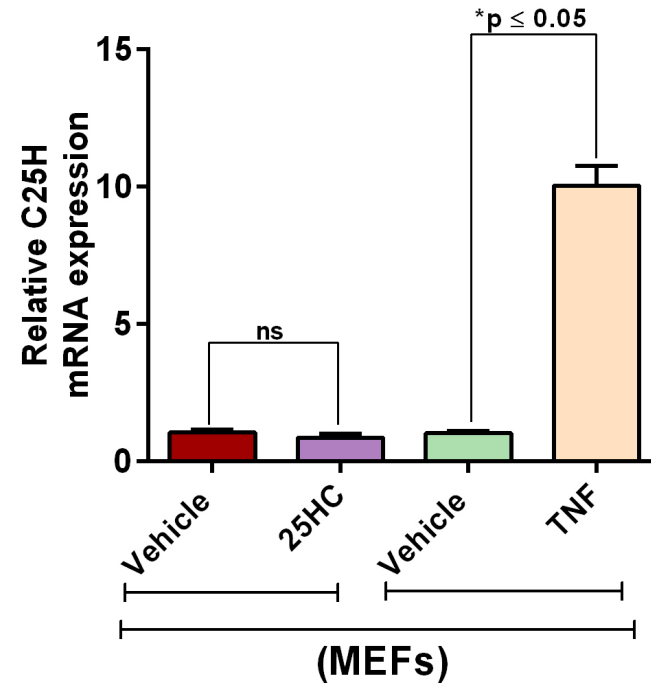

Supplement: S1 Fig — (A) 25HC production from RAW264.7 macrophages treated with TNF (100ng/ml) for 8h were analyzed by ELISA (n = 9; three independent experiments). (B) MEFs treated with either 25HC (80 pmol/ml) or TNF (10ng/ml) for 4h were analyzed for C25H mRNA expression by RT-qPCR (n = 12; four independent experiments). ELISA data are shown as Mean ± SEM. *p ≤ 0.05 using a Student’s t-test. RT-qPCR data are shown as Mean ± SEM.*p ≤ 0.05 using one-way ANOVA multiple comparison test. (PDF) [file pone.0257576.s001.pdf]

## S2 Figure

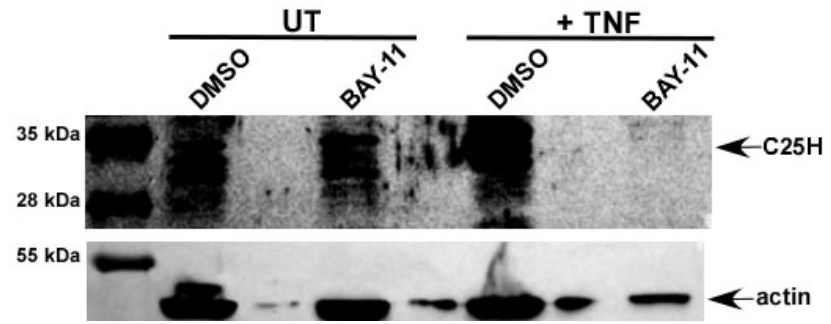

Supplement: S2 Fig — (PDF) [file pone.0257576.s002.pdf]
