# Supplementary material for: Tumor Necrosis Factor-alpha utilizes MAPK/NFκB pathways to induce cholesterol-25 hydroxylase for amplifying pro-inflammatory response via 25-hydroxycholesterol-integrin-FAK pathway
Source: PLoS One. 2021 Sep 22;16(9):e0257576. doi: 10.1371/journal.pone.0257576 (PMC8457477; doi:10.1371/journal.pone.0257576)

S1 Raw Images

Blots associated with Fig. 1c

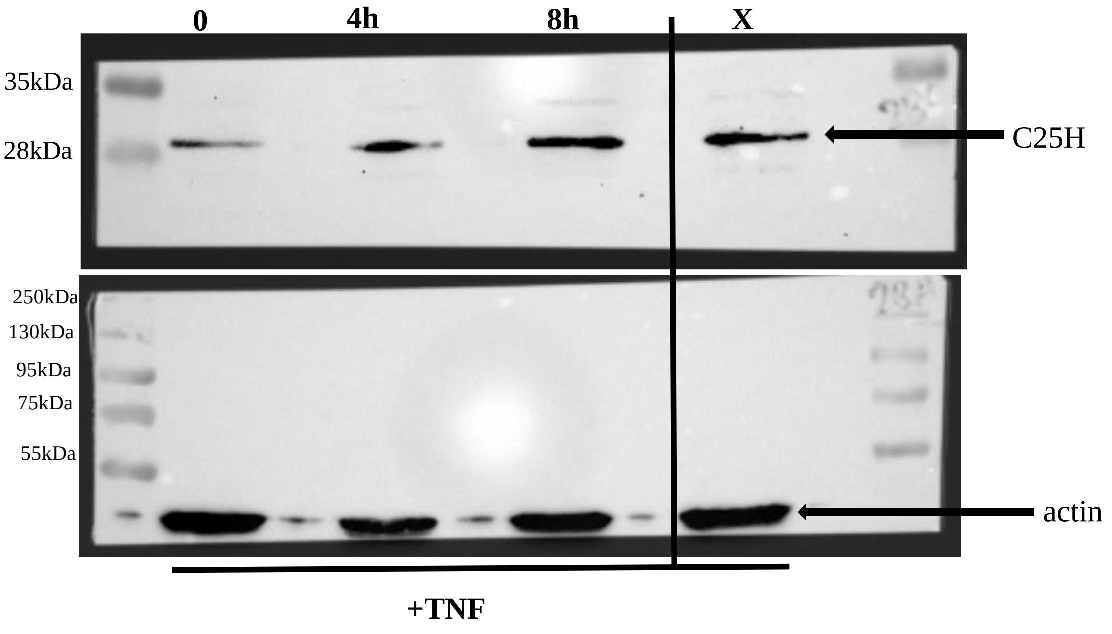

S1 Raw Images

Blots associated with Fig. 1e

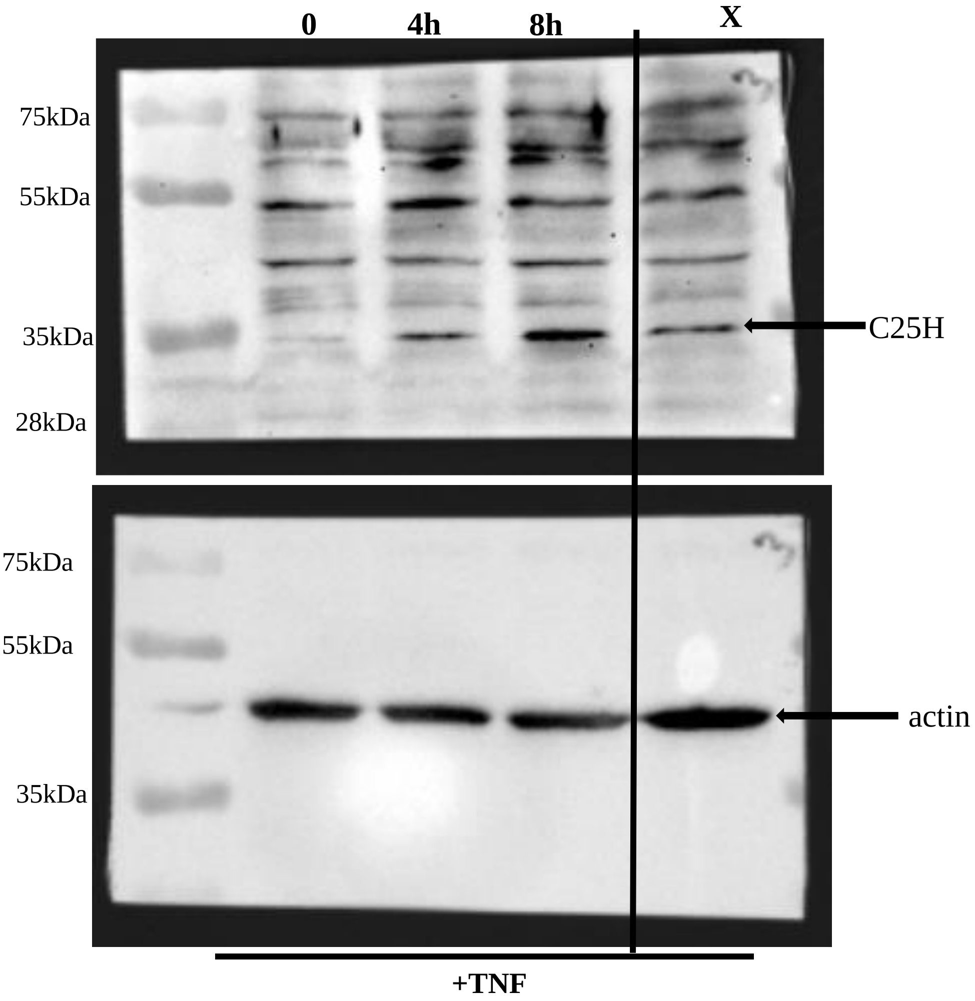

S1 Raw Images

Blots associated with Fig. 1g

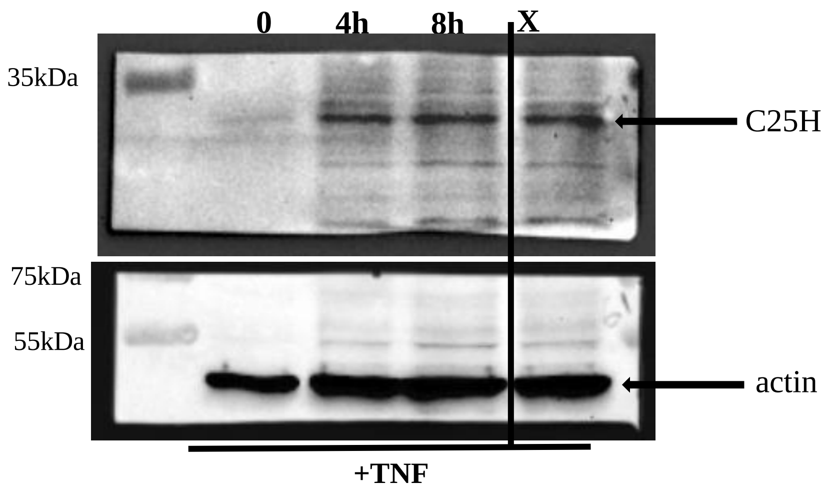

S1 Raw Images

Blots associated with Fig. 2b

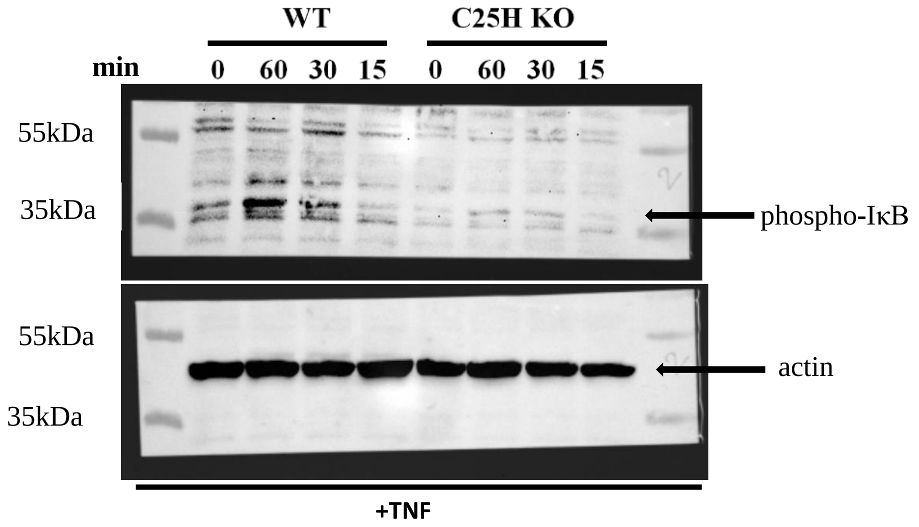

S1 Raw Images

Blots associated with Fig. 3a

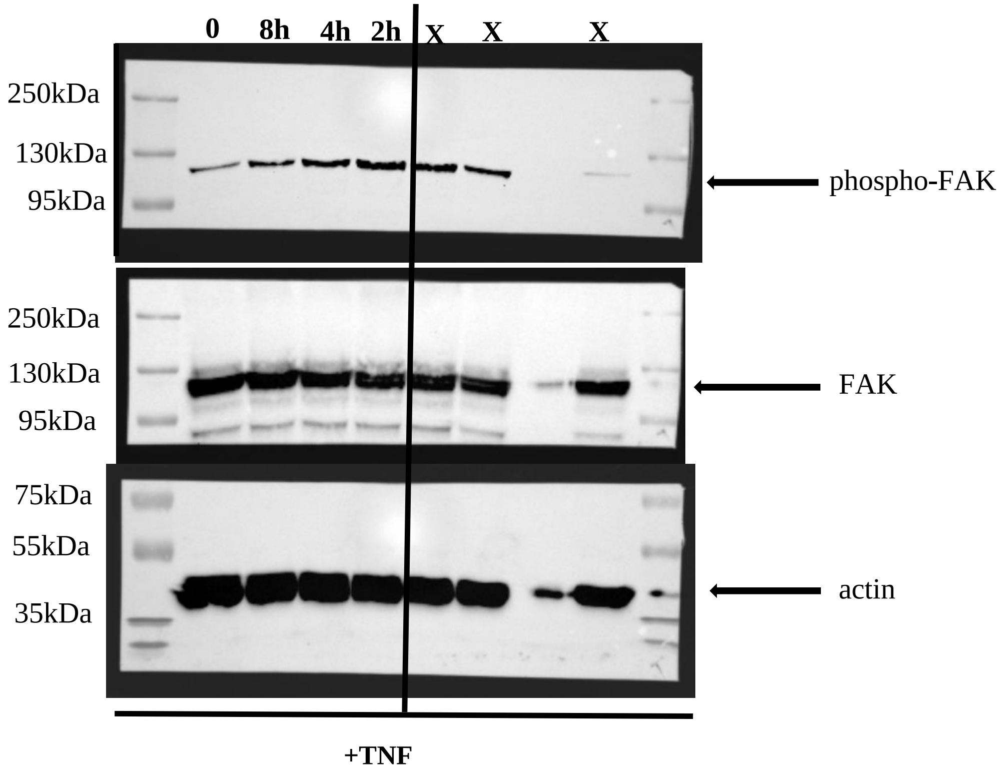

S1 Raw Images

Blots associated with Fig. 3e

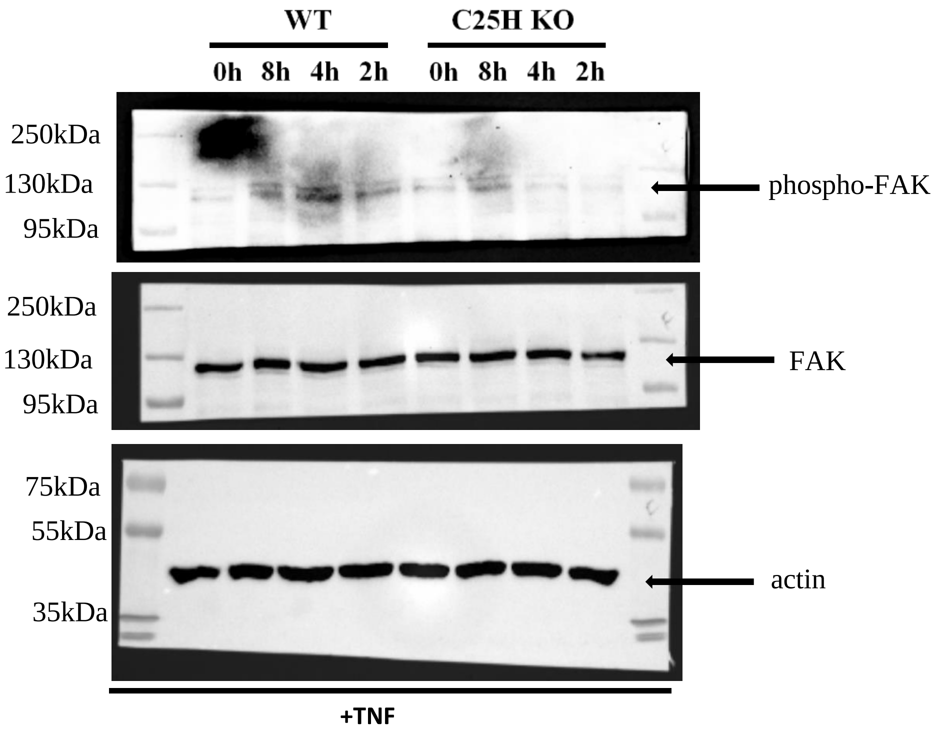

S1 Raw Images

Blots associated with Fig. 4b

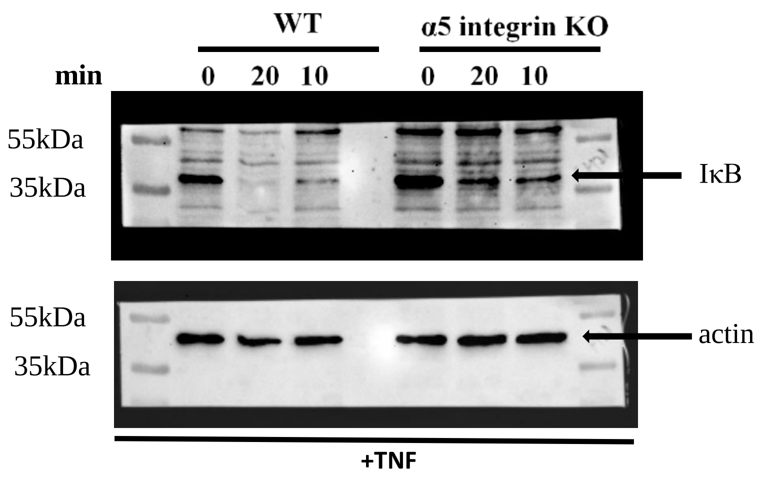

S1 Raw Images

Blots associated with Fig. 5b

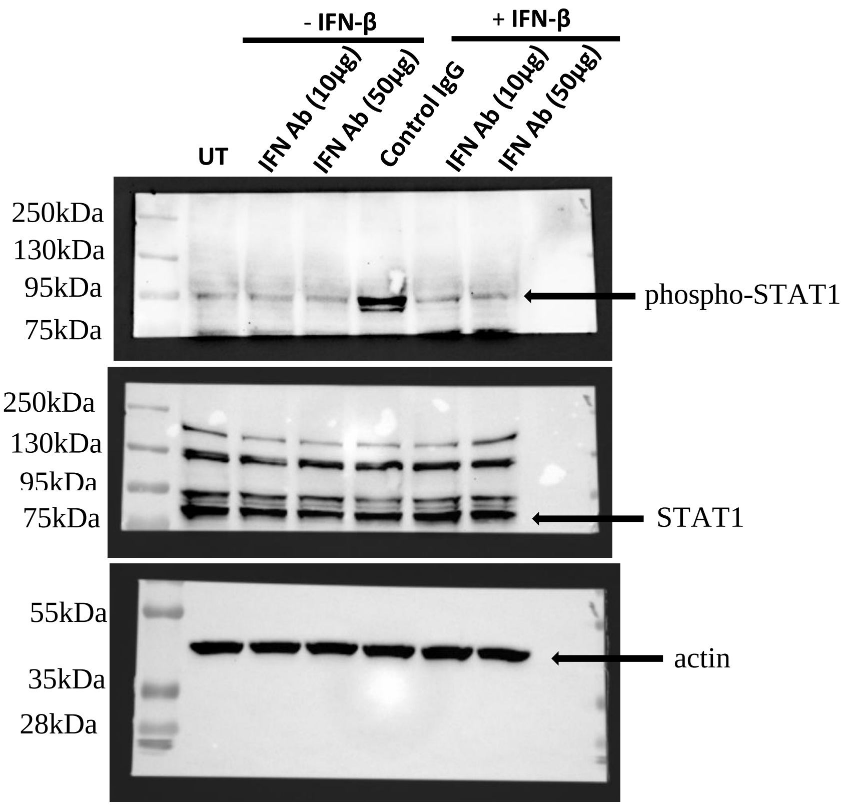

S1 Raw Images

Blots associated with Fig. 6e

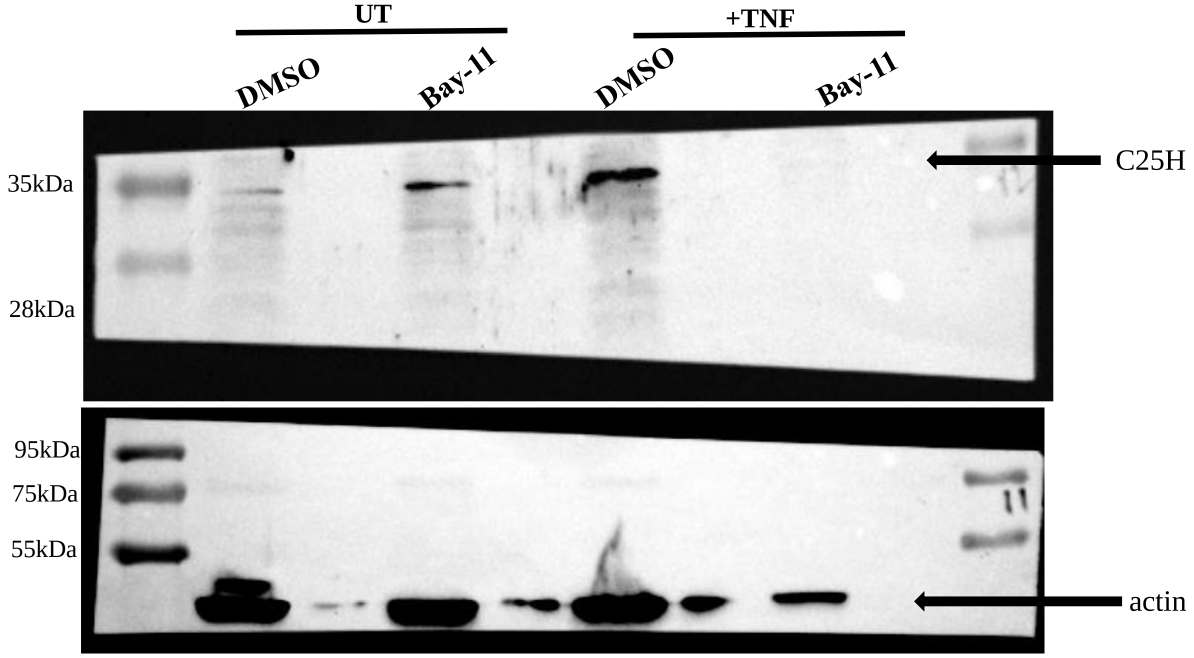

S1 Raw Images

Blots associated with Fig. 6g

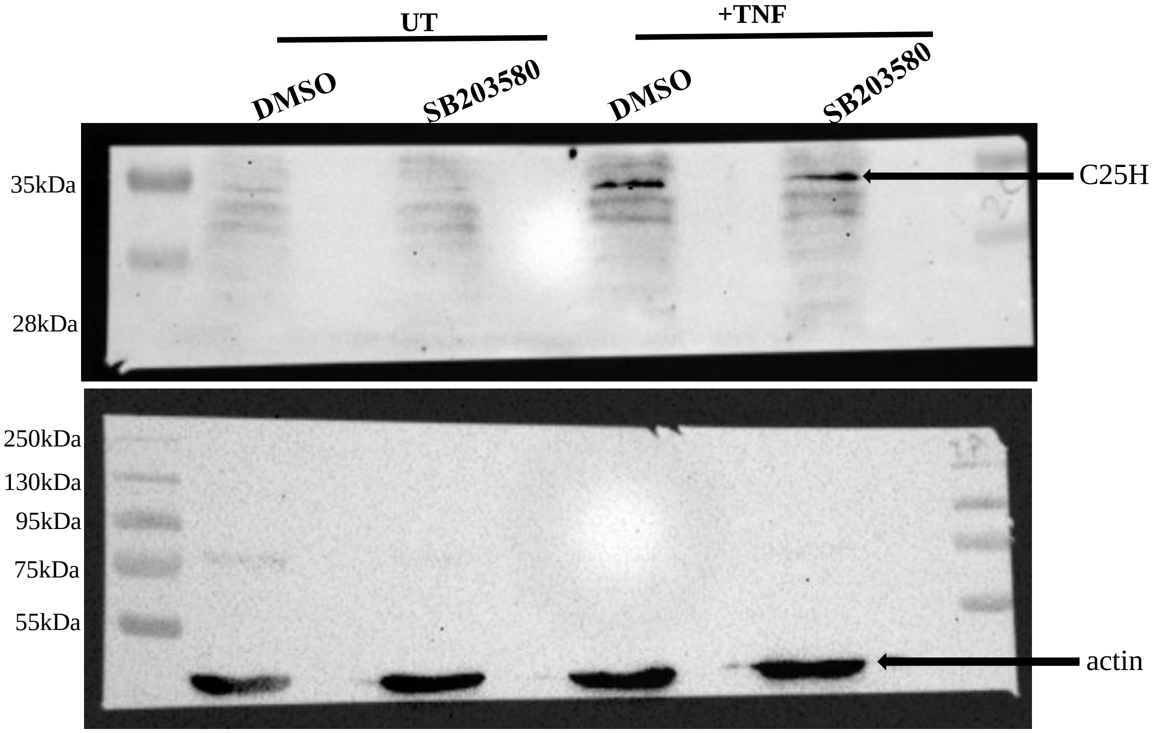

Supplement: S1 Raw images — (PDF) [file pone.0257576.s003.pdf]
